# The prion-like phenomenon in Alzheimer’s disease: Evidence of pathology transmission in humans

**DOI:** 10.1371/journal.ppat.1009004

**Published:** 2020-10-29

**Authors:** Ruben Gomez-Gutierrez, Rodrigo Morales

**Affiliations:** 1 Department of Neurology, McGovern Medical School, The University of Texas Health Science Center at Houston, Houston, Texas, United States of America; 2 Department of Cell Biology, Genetics and Physiology, Faculty of Sciences, University of Malaga, Malaga, Spain; 3 Department of Neuroscience, Baylor College of Medicine, Houston, Texas, United States of America; 4 Centro Integrativo de Biología y Química Aplicada (CIBQA), Universidad Bernardo O’Higgins, Santiago, Chile; University of Calgary, CANADA

## Prion propagation: A common mechanism among neurodegenerative proteinopathies

Most neurodegenerative diseases, such as Alzheimer’s disease (AD), Parkinson’s disease (PD), Huntington’s disease (HD), and prion diseases, share common pathogenic features. These include the presence of misfolded protein deposits and progressive neuronal loss in specific areas of the brain. Notably, the misfolded proteins involved in these diseases (prions, amyloid-β (Aβ), tau, and α-synuclein) share common structural, biological, and biochemical features, as well as similar mechanisms of aggregation and self-propagation. The infectious prion protein (PrP^Sc^) was the first disease-causing “proteinaceous infectious agent” ever described [1]. PrP^Sc^ has the ability to “transmit” its disease-associated conformation to normally folded prion proteins (PrP^C^). In turn, PrP^Sc^ can transfer its disease-causing information at different biological levels, including cell to cell, tissue to tissue, or between individuals. PrP^Sc^ particles associated with Creutzfeldt–Jakob disease (CJD) are able to transmit disease by different means, including corneal and dura transplants, implantation of electrodes, administration of cadaveric-derived human growth hormone (c-hGH), and blood transfusions [2].

Due to the striking similarities between PrP^Sc^ and other disease-associated protein aggregates, it is hypothesized that all of them have the ability to be transmissible. In the case of AD, Aβ and tau have shown to self-propagate both in vitro and in vivo, further supporting that pathological hallmarks of this disease can be transmitted. Remarkably, the growing evidence suggesting human iatrogenic transmission of Aβ pathology highlights the potential issue of interindividual transmission of AD-like neuropathology. In this manuscript, we discuss protein misfolding transmission mechanisms specifically focused on Aβ and the controversial hypothesis stating that some pathological features of AD might be transmissible.

## Prion-like propagation of Aβ pathology

Aβ, the peptide forming extracellular aggregates in AD brains, was described to self-propagate its misfolded conformation in vitro decades ago [3]. Further studies in a variety of platforms supported this particular property. One of them involved the intracerebral administration of AD brain extracts to young marmosets that displayed robust Aβ pathology 6 to 7 years later [4]. Taking advantage of transgenic animals mimicking some aspects of familial and sporadic AD, similar outcomes were obtained in considerable shorter times [5]. The central role of pre-formed Aβ aggregates (seeds) as inducers of brain amyloidosis was confirmed by several experiments showing that Aβ-depleted brain homogenates were not able to propagate Aβ pathology in AD transgenic mice [6,7] and others showing that intracerebral injections of purified synthetic aggregates were able to accelerate AD pathology [8]. Importantly, prion-like propagation of Aβ seeds can also occur when they are administered in the peritoneal cavity [9] or the blood stream [10] but not by other peripheral routes [11]. All these experimental evidences (reviewed in [12]) warrant further research to assess whether these prion-like transmission events are limited to intraindividual spread or can occur between individuals.

## Evidence of protein misfolding transmission in other neurodegenerative diseases

Prion transmission naturally occurs in different organisms besides mammals. These include yeast, fungus, bacteria, and plants. In these cases, prions are associated with adaptive functions for the host. This evidence suggests that prion transmission is a conserved mechanism across biological systems. Unfortunately, it seems that these events are in many cases associated with either disease progression or infection in the context of mammals (as observed in human and animal prion diseases).

Besides Aβ aggregates, many other disease-associated misfolded proteins have been experimentally shown to spread in a prion-like manner both in vitro and in vivo. Examples of these proteins are tau, α-synuclein, superoxide dismutase-1, serum amyloid-A (AA), and huntingtin. The misfolded version of some of these proteins have also been shown to propagate in a prion-like manner in humans. This is the case of α-synuclein, a hallmark protein involved in PD. In 2008, 2 independent studies demonstrated that different PD patients, who received transplantation of fetal mesencephalic dopaminergic neurons into the striatum, developed α-synuclein-positive Lewy bodies in the grafted neurons [13,14]. Similar findings have been documented in patients with HD that received fetal striatal transplants. In these cases, huntingtin protein aggregates were observed within the allografted neural tissue a decade after the transplants [15]. These observations shed light on the potential transmission of α-synucleinopathy and misfolded huntingtin in humans.

The previously mentioned evidence described prion-like transmission events occurring between cells and tissues but not bona fide interindividual infectious events as described for PrP^Sc^. The strongest evidence for prion-like infection to occur between individuals, outside of prion diseases, is found for a systemic amyloidosis involving AA in captive cheetahs (*Acinonyx jubatus*). AA amyloidosis is a leading cause of death in this animal species, and several reports demonstrate that increased animal density enhance the incidence and severity of this disease, as well as decrease its age of onset. Zhang and colleagues demonstrated that feces from captive cheetahs contain AA fibrils carrying high seeding activity and thus potential for interindividual transmission. Consequently, feces from diseased animals are proposed to be a vehicle for disease transmission similar to how it has been described for some animal prionopathies [16].

## Human-to-human transmission of Aβ amyloidosis

Despite the extensive evidence describing the prion-like properties of Aβ in animal models, evidence of this occurring in humans is controversial and have sparsely been reported (summarized in Table 1). One of the first studies that tackled the potential horizontal transmission of Aβ pathology in humans was reported by Irwin and colleagues [17]. In this study, the authors revised the National Hormone and Pituitary Program (NHPP) cohort database to assess whether c-hGH preparations containing disease-associated Aβ transmitted AD hallmark pathology to recipients in a similar fashion as described for CJD. Outcomes from this study failed to find any significant evidence of human-to-human transmission of Aβ misfolding. Later studies by Jaunmuktane and colleagues found evidence for the transmission of Aβ pathology in c-hGH recipients [18]. Here, researchers performed postmortem brain analyses of a subgroup of patients afflicted by c-hGH-induced CJD. They found that 4 of the 8 patients comprising this group had extensive parenchymal Aβ deposition, and 3 patients displayed widespread cerebral amyloid angiopathy (CAA). Two other patients also presented with focal cortical Aβ deposits. Further studies by Jaunmuktane and colleagues corroborated that AD-like neuropathology was indeed caused by c-hGH preparations contaminated with Aβ. Specifically, the authors demonstrated that original batches of c-hGH received by their cohort of iatrogenic Creutzfeldt–Jakob disease (iCJD) patients had substantial levels of Aβ, and these materials were able to induce both CAA and parenchymal Aβ plaques in transgenic mice after intracerebral inoculation [19]. These results, which are opposite to the findings by Irwin and colleagues [17], could be explained by the different incubation periods of both cohorts (mean of 16.3 years (first treatment to death) versus 33 years (first treatment to disease onset)), among other reasons.

**Table 1:** Summary of studies reporting potential amyloid pathology transmission in humans.

Seeds source	Aβ pathology		Tau pathology	Co-pathology	Reference(s)
	Parenchymal	CAA			
c-hGH	4 + 2 focal + 1 in PrP plaque	3 + 1 focal	Absent	iCJD	Jaunmuktane et al. 2015 [18]
	12/33 patients	14/33 patients	Sparse pTau-positive neurites /Absence of NFTs	iCJD	Ritchie et al. 2017 [20]
	4/12 patients	2/12 patients		None	
	1/24 patient	1/24 patient	3/24 patients with NFTs	iCJD	Duyckaerts et al. 2018 [28]
	2/8 patients	3/8 patients	NFTs and pTau-positive neurites	iCJD	Cali et al. 2018 [24]
Dura mater graft	13/16 patients (also in sCJD controls)	11/16 patients	11/16 patients (also in sCJD controls)	iCJD	Hamaguchi et al. 2016 [22]
	2/2 patients	2/2 patients	Absent	iCJD	Kovacs et al. 2016 [21]
	5/7 patients	5/7 patients	Absent	iCJD	Frontzek et al. 2016 [23]
	3/13 patients	8/13 patients	NFTs and pTau-positive neurites	iCJD	Cali et al. 2018 [24]
	1/1 patient	1/1 patient	NFTs and intracellular pTau	Cerebral hemorrhage	Herve et al. 2018 [25]
	2/2 patients	1/1 patient*	Absent	Cerebral hemorrhage and seizure	Banerjee et al. 2019 [26]
Tumor embolization with dura mater extract	1/1 patient	1/1 patient	Absent	Cerebral hemorrhage and seizure	Case 2 in Banerjee et al. 2019 [26]
Surgical instruments	3/4 patients	4/4 patients	Neuropil threads (1/4) and NFTs (1/4)	Cerebral hemorrhage	Jaunmuktane et al. 2018 [27]**
	1/1 patient	1/1 patient	pTau-positive neurites	Cerebral hemorrhage	Giaccone et al. 2019 [29]***

* In Case 3, Aβ pathology was analyzed only by 18F-Florbetapir amyloid PET imaging and had widespread cortical amyloid deposition. This might be the result of vascular amyloid deposition. In this table, this case was considered as parenchymal pathology and not included in the CAA count.

** In this study, no dural grafts were used for 1 out of 4 patients, whereas there is no information on grafts for the remaining 3 patients.

*** In this study, authors did not receive information allowing to confirm or exclude the use of cadaveric dura mater graft during the surgical procedure.

Aβ, amyloid-β; CAA, cerebral amyloid angiopathy; c-hGH, cadaveric-derived human growth hormone; iCJD, iatrogenic Creutzfeldt–Jakob disease; NFTs, neurofibrillary tangles; PrP, prion protein; pTau, phosphorylated tau; sCJD, sporadic Creutzfeldt–Jakob disease.

Induction of parenchymal Aβ deposits and CAA in recipients of c-hGH who died from causes other than prion disease was also reported by other groups [20]. Fairly similar Aβ pathology with predominant CAA was also reported for individuals exposed to cadaveric dura mater either by dural graft or by tumor embolization with dural extracts [21–26]. Interestingly, early onset CAA pathology has been reported in patients that underwent neurosurgical procedures in their childhood, raising the possibility that CAA might be accidentally caused by contaminated surgical instruments [27] as observed for CJD. Regardless of these assumptions, extensive research in different settings is needed to establish or discard prion-like transmission events associated with non-PrP^Sc^ misfolded protein aggregates.

## AD transmission risk and public health implications

As discussed above, several reports suggest that Aβ pathology may be iatrogenically transmitted between humans, albeit in restricted circumstances. It is important to note that the human brain specimens analyzed in these studies did not present the full spectrum of AD neuropathology. For example, tauopathy was minimal or absent in most samples analyzed and only 1 study, performed in a French cohort of patients treated with c-hGH, reported intraneuronal tau deposits in 3 individuals [28]. Notably, the c-hGH preparations analyzed in Purro and colleagues [19] contained measurable levels of tau, and future studies should determine whether tau in those vials have in vivo seeding capabilities. The method of preparation of c-hGH also seemed to be critical because only patients treated with samples following the Hartree-modified Wilhelmi protocol (HWP) developed Aβ pathology. Considering that most of the brains analyzed presented a pattern of Aβ deposition with strong vascular tropism, lacked neurofibrillary tangles, and did not present progressive cognitive impairment, it is suggested that brain amyloidosis affecting these individuals was different to AD. However, these disparities might also be (at least partially) attributed to prion disease that caused patients to die at relatively younger ages.

The novel concept suggesting that Aβ pathology is potentially transmissible in humans is relevant and warrants further research. However, it is worth mentioning that most of the cases described above underwent procedures that have not been used for decades. At this time, there is no evidence demonstrating that AD is contagious. In the same line, whether transmission of Aβ or tau misfolding lead to bone fide AD should be carefully investigated. However, potential procedures that might facilitate these events should be revised. For example, protocols to ensure the complete removal of misfolded proteins (seeds) from surgical instruments by nonstandard decontamination methods should be considered.
